# Virtual image of a hand displaced in space influences action performance of the real hand

**DOI:** 10.1038/s41598-020-66348-4

**Published:** 2020-06-11

**Authors:** Elisabetta Ambron, Alexander Miller, Stephanie Connor, H. Branch Coslett

**Affiliations:** 1 Laboratory for Cognition and Neural Stimulation, Dept. of Neurology, Perelman School of Medicine at the University of Pennsylvania, Philadelphia, USA; 2 Department of Psychological and Brain Sciences, University of Delaware, Newark, USA; 3 Neurology VR Laboratory, Dept. of Neurology, Perelman School of Medicine at the University of Pennsylvania, Philadelphia, USA

**Keywords:** Perception, Human behaviour

## Abstract

The rubber hand illusion (RHI) demonstrates that under some circumstances a fake hand can be regarded as part of one’s body; the RHI and related phenomena have been used to explore the flexibility of the body schema. Recent work has shown that a sense of embodiment may be generated by virtual reality (VR). In a series of experiments, we used VR to assess the effects of the displacement of the virtual image of subjects’ hands on action. Specifically, we tested whether spatial and temporal parameters of action change when participants perform a reaching movement towards the location of their virtual hand, the position of which was distorted on some trials. In different experiments, participants were sometimes provided with incorrect visual feedback regarding the position of the to-be-touched hand (Experiment 1), were deprived of visual feedback regarding the position of the reaching hand when acting (Experiment 2) or reached with the hand, the apparent position of which had been manipulated (Experiment 3). The effect was greatest when participants reached towards (Experiment 1) or with (Experiment 3) the displaced hand when the hand was visible during the reaching, but not when the vision of the hand was removed during the action (Experiment 2). Taken together, these data suggest that visual images of one’s hand presented in VR influence the body schema and action performance.

## Introduction

The mental representation of our bodies is not static but surprisingly – at least according to traditional conceptions – malleable. One influential illustration of this plasticity is provided by the well-known rubber hand illusion (RHI)^1^. To elicit this illusion, a rubber hand is placed in an anatomically plausible position in front of participants, while their hand is hidden from view. Simply synchronously touching the viewed rubber hand and the participants’ hidden hand causes many participants to judge the location of their hand to be displaced towards the rubber hand and to develop a sense of ownership of the rubber hand^1^.

As synchronized touch of the rubber and real hands is crucial to the illusion, the RHI is felt to strongly depend on the integration of tactile and visual information. Our brain solves the conflict between the spatial location of tactile stimulation (the real hand) and the visual information regarding hand position by integrating the two forms of information and assigning it to a visually defined location^2^. The illusion, however, is also crucially dependent on the participant’s mental representation of the body. Indeed, the illusion is not elicited using an object rather than the rubber hand^3^, if the rubber hand is placed in an anatomically implausible position^3^, or if the discrepancy between the locations of the rubber and real hands is excessive^4^. Because it is crucially dependent on features of one’s body representation, the RHI and related phenomena^2,3^ have been used to explore the flexibility of the body representation and specifically of the body schema.

The effects of the rubber hand are not limited to the sense of ownership but affect other senses or abilities as well. Studies have shown that when asked to estimate the absolute (“where is your finger?”)^3^ or relative (“estimate the distance between your finger and a visual stimulus”)^2,5^ position of their finger, participants show a systematic bias reflecting the fact that the apparent location of their hand is displaced toward the rubber hand, a phenomenon known as *proprioceptive drift*. Proprioceptive drift is considered to be an indirect measure of ownership of the rubber hand^6^ (but see^7^ for a dissenting view). Effects of the RHI have been reported in action by some investigators^1,8–10^; for example, Botvinick and Cohen^1^ asked subjects to reach with their unaffected hand to the hand which was stimulated to generate the RHI; their movement endpoints deviated toward the rubber hand location^1^. Effects of the RHI on action, however, have not been consistently reported^2,11^, raising the possibility of a dissociation between perception and action in the RHI and supporting the claim that action performance may be resistant to visual illusion^2,12^

At least 3 factors may influence the extent to which the RHI is evident in action. First, the effect of the RHI in action has been reported after both tactile stimulation^1,2,13^ and synchronous finger movements of the rubber and real hand^8,9^; action-based induction appears to more reliably induce the RHI in action^5^. Second, it is possible that the effect of the RHI in action might only be observed when the reaching movements are executed toward the stimulated hand^5^, rather than with the hand showing the diplacemement^2,11^. Third, most studies of the RHI in action have involved “open loop” movements in which visual information regarding target location is not provided. It is possible, however, that the effect of the RHI in action may be more apparent in “closed loop” movements during which vision of the hand is available during movement execution and the mismatch between visual and proprioceptive information is evident. Although the typical RHI paradigm does not permit one to test this possibility, virtual reality (VR) systems provide a unique opportunity to manipulate the relation between proprioception and vision.

Testing the effects of the RHI in action is important for several reasons. First, it addresses the still contentious question as to whether the action system is as sensitive as the perceptual system to visual illusion^12^. Second, it permits one to investigate the relationship between different body representations and other senses. Indeed, the observation of the RHI in perception but not action has been reported as evidence of dissociation between body image and body schema^2,14^. In this setting, body image refers to a static, largely semantic reflection of the body^2,14–16^ that has been postulated by some investigators to be involved in the perception of the RHI^2,5,11^. In contrast, the body schema is a dynamic representation of the form and posture of the body^16^, which articulates with motor systems in the genesis of action^2,14,16^. Evidence that the RHI does affect action performance has been used as evidence that proprioception has a more prominent weight than vision in defining the body schema^2,14^. The work reported here will contribute to the understanding of the relative contribution of proprioception and vision in the genesis of the body schema.

The RHI has been replicated using VR^17,18^ with either tactile^18–20^ or visuomotor synchronization of the VR and real hand^6,19,21^; the relative contributions of vision and proprioception in the RHI in action, however, have not been investigated. We addressed this issue in a series of 3 experiments that differed in important ways from previous work. First, we tested the effects of the RHI using a different paradigm than classical studies in which the hand is typically displaced medially and at large distance (14 cm or above) from the real hand^1,3^. In line with previous work exploring the RHI in action^8^, the rubber hand was displaced with respect to the location of the real hand in the vertical dimension but at a lesser distance compared to classical studies. Second, our studies are novel in that we used immersive, high definition VR (Vive, HTC) to assess movement time (MT) and action endpoint when reaching toward or with a hand that was visually displaced.

**Figure 1:**
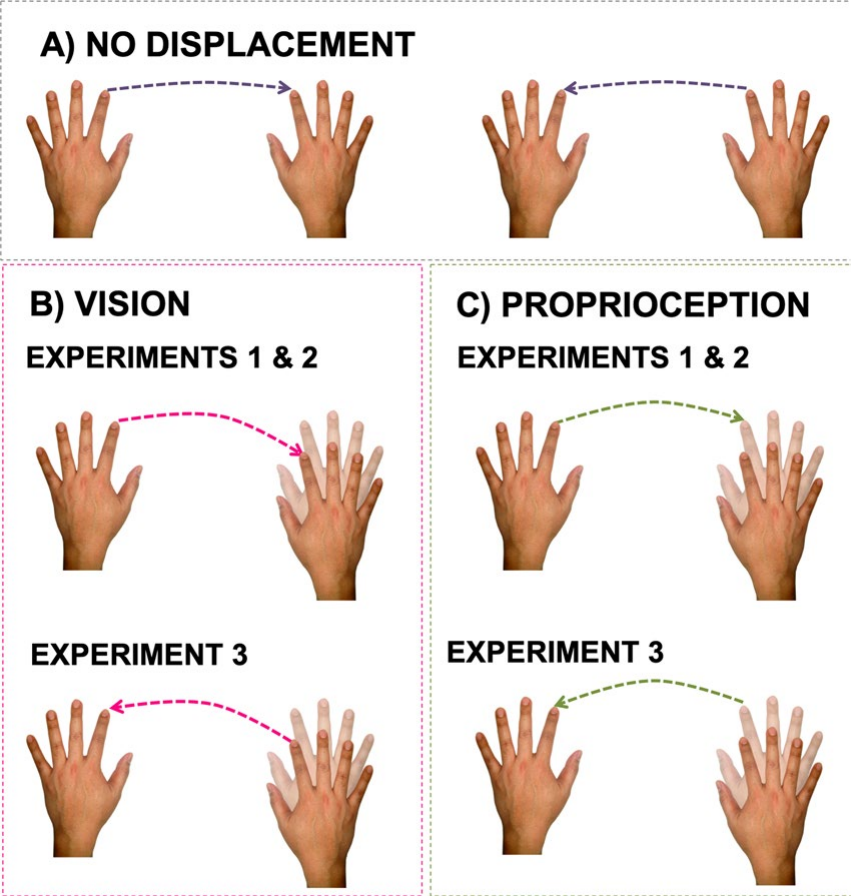
Panel A (NO DISPLACEMENT) depicts the expected movement trajectories (dotted lines) when the VR hand spatially overlapped with the real hand; in the left panel, the left index finger is moved to touch the right index finger whereas in the right panel the right index finger moves to touch the left index finger. In panel B (VISION) and C (PROPRIOCEPTION), the depicted movement trajectories are those that would be observed if subjects relied on vision or proprioception, respectively. In panels B and C, the lighter hand outline represents the veridical position of the subject’s hand on displacement trials whereas the darker hand represents the hand position as defined by vision.

In all experiments, participants played a card game during which the apparent position of one hand was gradually displaced in some trials; participants were not informed about the displacement. Participants reached with the index finger of one hand (non-target hand) towards the index finger of the other hand (target hand). In Experiment 1, participants moved toward the displaced VR hand while viewing both hands; on some trials, the VR input conveyed accurate information regarding the location of the hand whereas on other trials, target hand position was altered so that it appeared above or below the position of the real hand. In Experiment 2, we tested the effect of on-line visual feedback regarding the position of the reaching hand by asking subjects to reach in the absence of visual feedback regarding the position of the reaching hand. Finally, in Experiment 3, the visual image of the reaching hand was displaced, while accurate information about the location of the target hand was provided. In all the experiments, the magnitude of the hand displacement (7 or 14 cm) was varied to determine if the distance between the real and visual image of the hand further modulated action execution. If visual information regarding position of the virtual hands is incorporated in the neural representation of the body that generates action, one would predict an effect of hand displacement on reaching endpoint and MT for both the target (Experiment 1) and reaching hand (Experiment 3) (see Fig. 1). In contrast, as depriving participants of visual information would encourage reliance on proprioceptive information that is not directly manipulated via VR, one would expect participants to achieve normal or near-normal performance in both the displaced and not displaced conditions in Experiment 2 (see Fig. 1).

## Results Experiment 1

In Experiment 1, we assessed the effect of visual displacement of the hand on movement endpoint deviation and movement times. Both analyses employed Linear Multilevel Models (LMM), in which we compared a model composed of only random effects against a model which included the effect of the factor magnitude of the drift (−14, −7, 0, 7, 14 cm) in addition to random effects. The two models (basic and with displacement) were compared using the ANOVA function in R and significant results indicated a significant modulation of the performance as a function of displacement. In addition, for every subject we computed a best-fit slope of the endpoint deviation against the VR hand displacement and tested whether these slopes differed from zero.

### Endpoint Deviation

**Figure 2:**
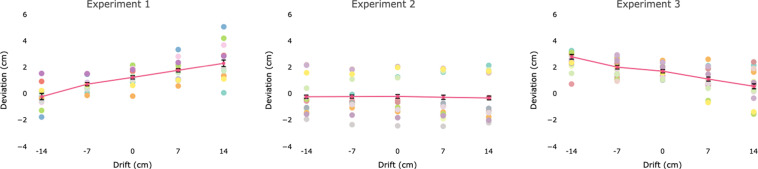
Deviation (computed as deviation in the y-axis of the endpoint of the moving hand from the target hand) across displacement magnitude in the three experiments. Positive values indicate upward deviation and negative values downward displacement towards the displaced hand in Experiments 1 and 2 and with respect to the non-displaced hand in Experiment 3. The error bars indicate standard errors of the mean. Individual participant’s performance for each displacement is represented with circular markers of different colors.

The endpoint of the action changed as a function of the visual position of the hand (see Fig. 2). In the no displacement condition, the endpoint of the action was shifted upward (*M* = 1.22 cm, *SE* = 0.13). As predicted, a larger upward deviation was noted when the target hand was displaced upward (that is, was visually perceived as being higher than it was) (7 cm: *M* = 1.76 cm/7.7% proportional displacement^1^^1^, *SE* = 0.12; 14 cm: *M* = 2.31/7.7% proportional displacement, SE = 0.25), while a downward deviation was observed when the displaced hand was shifted downward (−7 cm: *M* = 0.70 cm/7.4% proportional displacement, *SE* = 0.12; 14: M = −0.25/10.5% proportional displacement, SE = 0.24).

LMM showed that the direction of the drift contributed significantly to the model fit (logLik = −1507, χ^2^ (4) = 112, *p* < 0.001). Indeed, both downward (−14 cm: *t*_663_ = −5.92, *p* < 0.001; −7 cm: *t*_667_ = −2.23, *p* = 0.02) and upward drift (14 cm: *t*_666_ = 4.46, *p* < 0.001; 7 cm: *t*_666_ = 1.91, *p* = 0.056) differed from the no-drift condition. Furthermore, a larger deviation was observed for the 14 cm drifts as compared to the 7 cm drifts (*t* > 2.5, *p* < 0.05 in both comparisons). The significant effect of the drift was also confirmed in the analysis of the slopes. Indeed, we observed a significant difference from zero for the deviation (*t*_11_ = 4.74, *p* = 0.001).

### MTs

**Figure 3:**
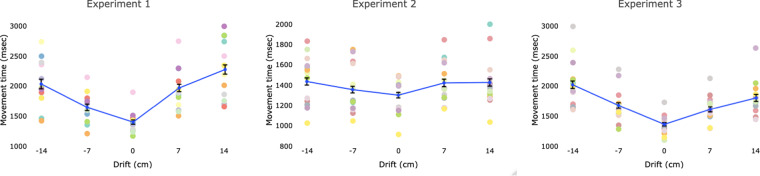
Average movement times across hand drifting conditions in the three experiments. The error bars indicate standard errors of the mean. Participants’ performance across displacement conditions is represented with circular markers of different colors.

Direction of displacement also influenced MTs (see Fig. 3). LMM showed that a model including the displacement direction better accounted for MT data than the random effects model (logLik = −5497, χ^2^ (4) = 132, *p* < 0.001). Participants took longer to perform the movement when the hand was displaced either upward (7 cm: *M* = 1965 ms, *SE* = 61, *t*_664_ = 6.87, *p* < 0.001; 14 cm: *M* = 2275, *SE* = 78, *t*_663_ = 11.13, *p* < 0.001) or downward (−7 cm: *M* = 1639 ms, *SE* = 54, *t*_664_ = 3.11, *p* = 0.002; −14 cm: *M* = 2032, *SE* = 81, *t*_661_ = 7.54, *p* < 0.001) as compared to the no displacement condition (*M* = 1390 ms, *SE* = 36). Furthermore, there was a significant difference between all the displacement conditions (p < 0.001), except for the comparison between −14 and 7 cm drift (*t*_666_ = 0.56, *p* = 0.57).

## Comment Experiment 1

Altering the apparent position of the target hand with VR influenced both movement trajectory and movement time. Indeed, deviation magnitude varied depending on whether the target hand was displaced upward or downward: it was higher with an upward drift and lower with a downward drift with respect to the no-drift condition, demonstrating that the endpoint of the movement trajectory was influenced by the location of the visual image of the target hand. Similar effects were noted in the MT data: MTs were longer in the drift as compared to no drift condition and were further modulated by the direction and magnitude of the drift. Taken together, data from the endpoint deviation and MT analyses suggest that vision of a virtual hand that is inconsistent with proprioceptive input regarding hand position alters participants’ hand movements. Our findings contrast with previous studies that failed to demonstrate an effect of the RHI in action, whether induced by means of sensory^2^ or motor^11^ manipulation.

Our study differed from several previous investigations in that in this study, vision of both the target and reaching hands was available throughout the task. It is possible that the RHI effect on action can be observed only in visually guided reaching movement.

The fact that participants’ reaching behavior was significantly (but modestly) influenced by inaccurate visual information suggests that the motor system is driven by a multimodal representation of hand position that incorporates both visual and proprioceptive/postural information. Under certain circumstances, the relative contribution of the two sources of input may be differentially weighted; participants with de-afferented limbs^16^ rely exclusively on visual information whereas for normal individuals, reaching in the dark necessitates a reliance on proprioception/postural information. Although Experiment 1 demonstrated that inaccurate visual information altered visually guided reaching performance, one might speculate that in the absence of visual information, participants rely on proprioceptive information, thereby reducing effects of the inaccurate visual input regarding initial hand position. In Experiment 2, we explored this issue by using the same induction phase that demonstrated reaching error in Experiment 1 but occluding vision of the hand during the reaching movement.

## Results Experiment 2

As for Experiment 1, data were analyzed using LMM on both endpoint deviation and movement time. Furthermore, for the deviation we also computed the slope against the VR hand displacement and computed a one sample t-test against zero.

### Endpoint Deviation

The movement endpoint was not influenced by drift direction or magnitude (see Fig. 2). LMM analysis showed that the random effects model predicted performance as well as the model including displacement direction as a fixed factor (logLik = −1266.7, χ^2^ (4) = 1.38, *p* = 0.84). The analysis of the slopes confirmed a non-significant effect of the hand displacement on deviation (*t*_13_ = −0.32, *p* = 0.75) (see Fig. 2).

### MT

An effect of the manipulation of the VR hand position was observed for MT (see Fig. 3). The model including displacement direction was a better predictor of MT data than the random effects model (logLik = −6162, χ^2^ (4) = 13.2, *p* = 0.009). MTs were faster in the no displacement condition than all displacement conditions, except for the −7 cm drift (*t*_808_ = 1.07, *p* = 0.28). No significant difference was observed between the displacement conditions (*t* < 1.9, *p* > 0.06 in all comparisons).

## Comment Experiment 2

The effect of visual displacement of the VR hand in action performance observed in Experiment 1 was replicated in the MT analysis but not in endpoint deviation. One possible explanation for the less robust effect of the experimental manipulation is that when vision of the hand is removed, participants increasingly rely on proprioceptive and postural information^2^. Our findings contrast with previous studies showing a proprioceptive drift in pointing movement without vision of the hand after movement-based induction of the RHI^5,8^. In these studies, participants performed reaching movements towards the finger that was subjected to the RHI in open loop conditions, without the visual feedback of the hand. A major difference between these studies and the present study is in the duration of the induction phase, which was approximately 3 minutes in previous work^5,8^ but only 30 seconds in the present study. The short time of our induction phase might not have been sufficient to induce long lasting effects on proprioception. However, the effect of the drift on MTs also suggests that some effects of inaccurate visual information remained when vision of the hand was occluded, demonstrating a subtle effect of previous inaccurate vision when action execution relied only on proprioceptive information. A dissociation between temporal and spatial parameters of action is not unprecedented. We observed this dissociation in a study in which subjects viewed their hand and the target while wearing magnifying glasses, but vision of the hand was prevented during the reaching movement. MTs changed when reaching for a normal or magnified object without the vision of the hand, whereas the grasping parameter remained consistent^22^.

We attributed the deviation toward the displaced hand demonstrated in Experiment 1 to the fact that visual information about hand position influenced the mental representation of the location of the displaced hand, at least partially over-riding proprioceptive inputs. There is a substantial literature, however, demonstrating that reaching movements rapidly adjust and follow changes in the visual position of any target^23,24^. Thus, an alternative possibility is that the reaching errors documented in Experiment 1 are not specific to the participants’ body part but would have been observed when reaching to any type of target. To test whether our VR manipulation induces a change in the on-line mental representation of the body, we performed Experiment 3, in which participants reached with the displaced hand rather than to the displaced hand. We reasoned that if data from Experiment 1 reflected the “automatic pilot” previously reported^23^, one would not see an effect of displacing the reaching hand because the motor system would automatically adapt to the visually defined target; in contrast, if displacing the hand caused an erroneous updating of the mental representation of the body in space, one would expect to observe systematic errors in reach kinematics reflecting the incorrect starting position of the reaching hand induced by the VR manipulation.

Experiment 3 addressed a second issue as well. It remains unclear whether the effect of the RHI in action can be observed when performing the action with the hand receiving the RHI. Although evidence of the RHI in action has been reported for the hand receiving the RHI by some investigators^25^, this has not been consistently observed^2,10^^.^

We investigated these issues by inducing a mismatch between vision and proprioception for the hand performing the reaching movements. In this experiment, participants were asked to perform reaching movements with a veridical or displaced virtual hand. In line with previous work^25^, we predicted the reaching deviation to be in the opposite direction from that observed in Experiment 1; that is, if the drift induces an upward shift in the perceived position of the reaching hand, participants would reach below the target because they would compute a movement trajectory that includes a greater downward component. Thus, the endpoint would be below the target hand with upward drift and above the target hand in the downward drift condition (see Fig. 1). In contrast, if data from Experiment 1 reflect a general effect of the adaptation to changes in the visual location of the target, one would not expect to observe changes in kinematics when the displaced hand performs the action.

## Results Experiment 3

### Endpoint Deviation

Displacement direction contributed significantly to the model fit for reach deviation (logLik = −1673, χ^2^ (4) = 119.9, *p* < 0.001). In contrast to Experiment 1, we observed an upward deviation of endpoint; displacement was significantly higher in the −14 cm condition (*M* = 2.78 cm /7.9% proportional displacement, *SE* = 0.17) as compared to the 0 cm displacement condition (*M* = 1.68 cm, *SE* = 0.12) (*t*_788_ = 5.06, *p* < 0.001) and upward displacement conditions (7 cm: *M* = 1.08 cm/8.6% proportional displacement, *SE* = 0.15, *t*_792_ = −2.8, *p* = 0.004; 14 cm: *M* = 0.53/7.8% proportional displacement, *SE* = 0.18, *t*_788_ = −5.55, *p* < 0.001). Upward deviation was observed in the −7 cm displacement condition, but this did not differ from the no displacement condition (*M* = 1.99 cm/4.4% proportional displacement, *SE* = 0.17, *t*_788_ = 1.20, *p* = 0.22). Furthermore, the analysis of the slope confirmed a significant difference from zero for the deviation (*t*_13_ = −4.86, p < 0.001).

### MT

As in Experiments 1 and 2, the model including displacement direction accounted for the overall MT data better than the random effects model (logLik = −6329, χ^2^(4) = 102, *p* < 0.001). MTs were longer in the upward (7 cm: *M* = 1605 ms, *SE* = 42, 14 cm: *M* = 1798, *SE* = 58) and downward (−7 cm: *M* = 1672 ms, *SE* = 44, 14 cm: *M* = 2019, *SE* = 62) drift condition with respect to the no-drift condition (*M* = 1353 ms, *SE* = 28) (*t* > 3.4, *p* < 0.001 in all comparisons). Furthermore, longer MTs were noted on the 14 cm displacement condition as compared to the 7 cm condition (*t* > 2.5, *p* < 0.01 in both comparisons).

## Comment Experiment 3

In line with our predictions, we observed an effect of inaccurate visual information on both deviation and MTs. In particular, the endpoint of the reaching movement was shifted downward when the VR hand was gradually displaced upward, and shifted upward when the VR hand was displaced downward. This evidence is in line with a previous study showing the effects of the RHI during action execution. Indeed, Newport *et al*.^25^. noted a similar modification of movement trajectories when performing a reaching movement of the hand that had been subjected to the RHI manipulation in an open loop condition (i.e. in which the vision of the hand was prevented during action performance). The deviation of the movement trajectory of the hand was interpreted as evidence that the rubber hand was incorporated into the body schema, so that action performance was adjusted to the visual image of the hand. Our findings are consistent with this interpretation.

Contrary to Experiment 1, we observed an effect of the magnitude of the shift for both deviation and MTs, with the misplacement of the endpoint being larger and MTs longer in the 14 cm than 7 cm hand drift condition. This evidence, in conjunction with the fact that the magnitude of the deviation was greater in Experiment 3 as compared to Experiment 1, suggests that the visual image of the hand is incorporated in action performance to a greater extent in Experiment 3 as compared to Experiment 1.

Taken together these data suggest that visual information regarding the location of participants’ hands, even when inaccurate, altered motor performance in a fashion that suggested that the virtual hand influenced action planning for the real hand.

### Perceived ownership of the VR hand

**Figure 4:**
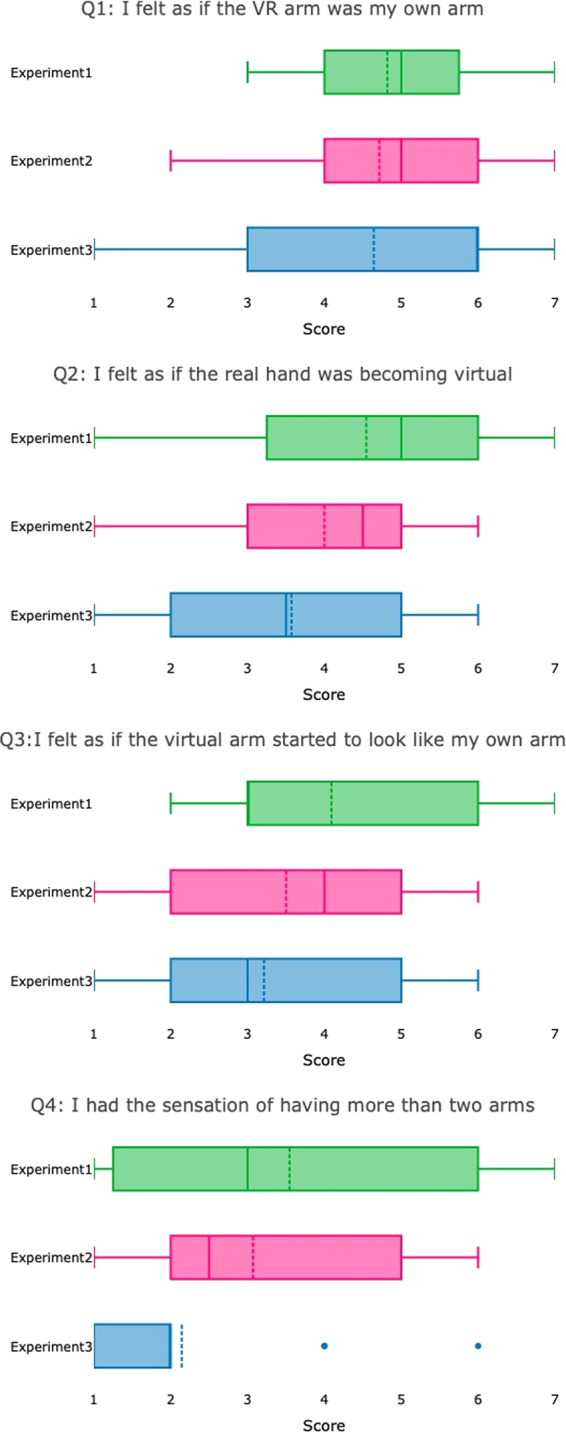
Participants’ mean (dotted line) responses to the questionnaire about ownership of the VR hand for the three experiments. The lines within each box represent the first interquartile, the median and the third interquartile. Whiskers represent the minimum and maximum; dotted lines represent the mean and SD. The plots were created using plotly.

Results of the questionnaires regarding the sense of ownership of the VR hand are reported in Fig. 4. Across experiments, participants reported a sense of ownership of the VR hand around the middle score.

## Discussion

These studies demonstrate that a virtual hand controlled by the participant influenced motor performance of the participant’s real hand. These effects encompass both temporal (MTs) and spatial (deviation) aspects of action and are evident when the vision of the hand is available during the reaching movements (Experiment 1 and 3) and when the action is executed with the displaced hand (Experiment 3).

Our results are important in several respects. First, this study informs the discussion regarding the dissociation between perception and action in the RHI^2^. Although data from some studies suggest that action is resistant to the RHI^2,12^, we demonstrate that illusory visual feedback regarding hand position modulates on-line reaching behavior.

Second, the present work provides evidence on the body schema and action performance. Previous work has shown that changes in the body form (e.g. perceived size^22,26–28^) influence action; the present work expands these observations upon the relationship between visual input and body representations more generally. By inducing a mismatch between vision and proprioception, we induce systematic deviation in reaching. We believe that this is likely to reflect a modification of the body schema, the online representation of the body in space derived from vision and proprioception, that articulates with spatial systems in the genesis of action^15^; these findings demonstrate that the body schema may be modified by just 30 seconds of distorted visual feedback. Since visually guided reaching movements strongly rely on vision of the hand^29,30^, it is not surprising that the effects were greater when vision of the hand was available.

Third, the present results inform the debate regarding the relative weight of vision and proprioception in the body schema and in action.

Our results suggest that, when available, vision plays a crucial role in computing hand location and reaching trajectory. We note, however, that the interplay between vision and proprioceptive/postural inputs is complex; although we report significant effects of the visual manipulation across experiments, the magnitude of the deviations were small (maximal proportional displacement of 10.5% in Experiment 1 and 8.6% in Experiment 3) in relation to the magnitude of the visual perturbation (7 cm or 14 cm), clearly indicating that proprioceptive input remains relevant to the computation of hand position.

One potential explanation for the relatively modest impact of the visual displacement is provided by the fact that we implemented a visual displacement in the vertical dimension. Previous work has shown that the visual perturbation of target location induced by a mirror generates a reaching deviation that differs as a function of the direction of the perturbation^31,32^. In particular, Snijders, Holmes, & Spence^31^ observed a larger endpoint error when the visual target displacement occurred in the horizontal as compared to radial dimension. This effect was explained with the optimal integration model^32^, which proposes that vision plays a more prominent role in reaching movement in the azimuth/horizontal direction, while proprioception may be more prominent in reaching in depth. As in the present study, the visual displacement of the hand occurred in the vertical dimension, rather than in the horizontal dimension; as in many studies of the RHI, it is possible that the magnitude of our endpoint deviation was small due to the more prominent weight of proprioception along this dimension of the space. Future work should explore this issue further by comparing the effect of the displacement of the VR hand across horizontal and vertical dimensions in reaching movements.

In addition to these theoretical contributions, our work has important methodological implications. First, we were able to demonstrate consistent effects of visual input on action, a feat that has proven difficult in some studies of the RHI in action^6,17^. Using the combination of immersive VR and motion tracking, we were able to induce and assess the effects of altering visual input at the same time. Furthermore, our system allowed us to measure effects while the illusion was present. Indeed, the VR followed the motion of the participant’s real hand. In most studies to date, the effect of hand displacement on action is measured only after the illusion was induced and without participants having complete control of the VR hand. Second, we were able to induce and test the effect of the displacement online in the hand performing the action as well as the hand serving as the target of the action. Finally, in contrast to much previous work (e.g., 5, 7), we were able to induce a significant alteration in the apparent visual location of the hand with an induction phase of only 30 seconds, allowing for the acquisition of a far larger number of trials in an experimental session.

We note that the sense of ownership induced in the present study is not as strong as the one observed in the classical^1^ or VR^17^ studies with the RHI. One possible explanation is that after the execution of the reaching movement, the physical contact between the two hands might have provided participants with feedback regarding the visual and proprioceptive mismatch occurring in some trials, thereby reducing the sense of ownership. Another possibility is that the sense of ownership may have been reduced by a discrepancy between the visual representation of the extremity and the appearance (e.g., size, shape, color, presence of distinctive features, etc.) of the participant’s actual extremity. Unfortunately, we do not have information to determine if the ownership ratings were influenced by such factors. Future work should investigate the effect of race, ethnicity and gender on the embodiment of VR hands. Finally, as we did not control for participants’ level of suggestibility in the questionnaire, it is not possible to exclude the influence of this factor in the participants’ sense of VR hand ownership.

The present study has several limitations. One relates to the assessment of ownership of the VR hand. As the main focus of our work was the effect of the visual displacement of the VR hand on reaching movements of the real hand, the different conditions were presented in a random order and a general questionnaire of ownership was presented only at the end of each experiment. Furthermore, we did not inquire about participants’ awareness of the hand displacement during the execution of the trials. Another issue is that the starting point of the action was not defined, but depended on the location of the hand in space when the tone signaled participants to stop the card game. We used this approach to reduce participants’ information regarding the position of their body in space prior to starting the reaching movement; although this makes it difficult to compare MTs across trials and conditions, we reasoned that moving the hand to a standard starting point would have provided proprioceptive information that could have undermined the visual illusion. We note that MTs were longer than one might have expected. Several factors could be relevant. First, we considered online adjustments of the reaching movements to be part of the movement trajectory. Second, the lack of familiarity with reaching in a VR environment may have made participants tentative, thereby slowing MTs. Finally, a power analysis was not carried out before the study; we believe that the lack of effects of deviation in Experiment 2 is unlikely to be attributable to insufficient power, however, because Experiment 2 had a marginally greater number of subjects than Experiment 1 (14 vs 12) and there was very little effect of magnitude of deviation in Experiment 2.

## Material and Methods

The experimental manipulation varied across experiments but the equipment, data extraction and analysis, and the assessment of perceived ownership of the VR hand were consistent across experiments.

The Institutional Review Board of the University of Pennsylvania approved the present study; all the experiments were conducted in accordance with the Declaration of Helsinki (as of 2008) and all participants provided informed consent.

### Equipment

The VR environment was generated with the Unity3D Game Engine (Unity Technologies) and run with a GTX 1060 Graphics card. The environment was rendered with an Oculus Rift CV1. A Leap Motion attached to the Head Mounted Display tracked the pose and position of each hand. To record participants’ real movements in space, we used a 3D electromagnetic tracking system (trackSTAR, Ascension Technologies Inc., Burlington, Vermont), with 100 Hz sampling rate and two 6DOF electromagnetic sensors attached to participants’ index fingers using Velcro.

### Data extraction and analysis

Coordinates of each sensor were filtered using a second-order Butterworth filter, with a cut-off frequency of 10 Hz. For each trial, the movement’s trajectories and velocity profiles were visually inspected, and the examiner manually identified the onset and offset of the action. Movement onset was identified as the point in which the velocity profile increased approximately 5% of the peak velocity. Similarly, the offset was defined as the time at which the velocity profile decreased to 5% of the peak velocity and the target hand was reached (distance between the hands reached a plateau). This less conservative threshold has been used in several previous works^26,33–35^ and was used to capture the endpoint of the action where the target hand was reached. Online corrections were included in the data extraction. Customized programs written in R and Labview (National Instruments) were used to extract kinematic parameters.

There were 2 dependent variables: movement endpoint relative to the target and movement time (MT). For each trial, we extracted movement coordinates of each marker (target and non-target hand) and computed the deviation between the endpoint of the reaching movement and the target. Therefore, deviation was computed by subtracting the y-coordinate of the endpoint of the marker placed on the moving hand from the y-coordinate of the endpoint of the marker placed on the target hand; positive values indicated upward deviation of the moving hand and negative values downward deviation of the moving hand. MTs reflected the time participants took to execute the movements, defined as the time between onset and offset of the action as described above. MTs were chosen as a measure of the temporal aspect of movement because this factor has been shown to be a sensitive measure of kinematic changes related to the effect of vision of the body^22^.

Extracted data were analyzed using multilevel modeling in R with random intercepts and random slopes of trials for subjects. In each experiment, we ran two different analyses. We compared a basic model, including only random effects, with a model including displacement (−14, −7, 0, 7, 14 cm) as a fixed factor. As it was not the focus of the present work and no specific predictions were formulated, we did not test for the possible effects of the laterality (right/left) of the hand performing the reaching movement. However, to control for its possible influence, this factor was included as a random factor in the analysis. Models were compared using the ANOVA function in R.

In addition, we computed a best-fit slope of the endpoint against the VR hand displacement (−14, −7, 0, 7, 14 cm) for each participant. These slope-values were then analyzed using a t-test against zero.

### Perceived ownership of the VR hand

To investigate the sense of ownership of the VR hand, participants were presented with the four-statement questionnaire developed by Yuan & Steed^30^ and were asked to rate their level of agreement with each statement using a Likert scale ranging from 1 (strongly disagree) to 7 (strongly agree). A rating of 4 indicated a neutral score. The statements were: (1) I felt as if the VR arm was my own arm; (2) I felt as if the real hand was becoming virtual; (3) I felt as if the virtual arm started to look like my own arm in some aspects; (4) I had the sensation of having more than two arms. Questions 1 and 2 were directly related to the illusion, while questions 3 and 4 were unrelated control questions^30^.

## Experiment 1

### Participants

Twelve young adults (7 females, age *M* = 24.72 and *SD* = 6.60) took part in this experiment.

### Task

Participants were asked to play a card matching game with their virtual hands in an immersive VR environment. The game started with an array of green cards presented in the frontal plane at a comfortable reaching distance. The task was to find 2 cards with the same number in the array. Participants started by touching a green card with one hand; this caused the card to turn over, revealing the face of a French playing card. Next, participants touched a different card, causing it to turn over. If the numbers matched, the card faces remained displayed. If they did not match, the cards flipped back to the green side and participants started the process again. Participants continued until they had identified all the matches in the array; when this was completed, a new set of cards was presented and the process was continued. Cards were presented in two columns. Participants’ virtual hands were visible throughout the card game. Participants reached to cards on the right side of the array with the right hand and cards on the left side of the display with the left hand.

**Figure 5:**
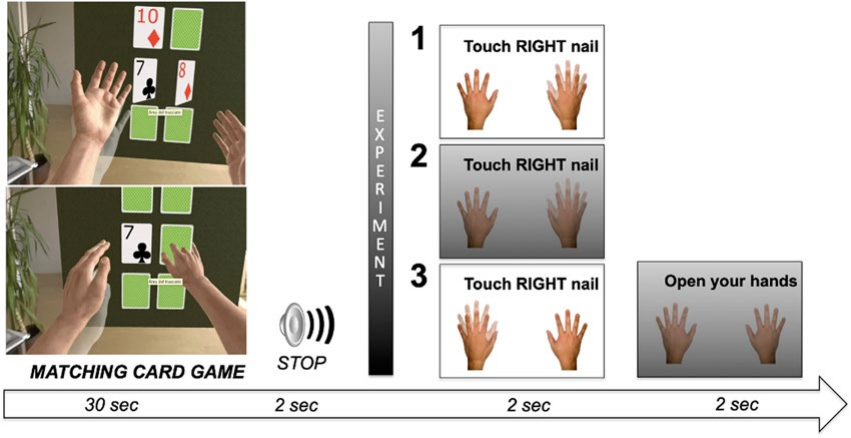
Depiction of a single trial. The matching card game, the stop signal and the end of the trial were common to all the experiments, but the presentation of the instructions and the following reaching task varied. In Experiment 1, instructions were displayed above the visible VR hands and the reaching was performed towards the displaced hand; in Experiment 2, instructions were displayed above a black screen and the vision of the hands was eliminated during the reaching. In Experiment 3, instructions were displayed above the visible VR hands and reaching was executed with the displaced hand. In all examples below, the VR hand is represented with a natural color, while the semi-transparent hand represents the position of the real hand that was not visible to the participants during the task.

As shown in Fig. 5, each trial started with participants playing the card game for 30 seconds, after which a tone (1000 Hz, 1 sec) signaled them to stop moving their hands and keep them stationary in their current location. After two seconds, written instructions were presented in the top half of the VR environment indicating which hand would serve as the target; participants then moved the non-target index finger to contact the tip of the index finger of the target hand. For example, on one trial “Touch the right nail” appeared; participants moved the left hand to touch the nail of the right index finger. As subjects initiated reaching movements from their location at the time of the tone, the distance between and relative locations of the hands varied across trials. The VR hands, which were always unclenched with the fingers comfortably separated, were visible during the execution of the movement. Participants were given two seconds to perform the movement, after which a black screen appeared, and participants moved their hands to a neutral position with their hands in front of them, separated by a few inches with the fingers spread and palms facing outward.

While visual input regarding the position of the non-target (reaching) hand position was accurate throughout, the position of the target hand was systematically distorted while participants played the card game. Ten conditions were presented in random order, defined by the reaching hand (left and right) and the visual displacement (downwards and upwards 7 and 14 cm respectively, or no displacement). There were total of 60 trials in one session. That is, on 20% of trials, information regarding the position of the target hand was veridical throughout whereas on 80% of trials, the position of the target hand was slowly displaced up or down by either 7 or 14 cm. The hand displacement started immediately after the onset of the trial during the card game, continued progressively for 30 seconds, and then persisted without change during the execution of the reaching movement. Participants were not informed about this drift during the course of the experiment.

To keep participants’ interest high, the game increased in difficulty during the experiment by an increase in the size of the card array from the initial 2 × 6 array to a maximum of a 7 × 7 array. The array size was increased after the participant matched all of the cards; each trial started with a 2 × 6 array.

## Experiment 2

### Participants

A different group of fourteen young adults (6 females, age *M* = 23.85; SD = 3.3) took part in this experiment.

### Task

Participants were presented with the same task as Experiment 1, with the only difference being that the vision of the hand was eliminated prior to execution of the reaching movement. As in Experiment 1, there were 60 trials presented in random order in which we manipulated the target hand (right, left), and displacement conditions (no displacement, upward displacement, downward displacement) and magnitude of the displacement (7 cm, 14 cm).

## Experiment 3

### Participants

A new group of fourteen young adults (9 females, age *M* = 23.5; SD = 4.4) took part in this experiment.

### Task

This experiment had exactly the same design, structure, trials and conditions of Experiment 1, except that the drift occurred in the reaching hand, following the same conditions of the previous experiments, while the target hand was not displaced.

## Data Availability

The datasets generated during and/or analyzed during the current study are available from the corresponding author on reasonable request.

## Footnotes

1 ^1^We first computed the difference of the endpoint deviation in the drift condition from the no-displacement condition, and then calculated the proportion of displacement by dividing the difference in the endpoint for each condition by the magnitude of the visual displacement in that condition (eg. ^8,15^).
